# Development of a computer-controlled closed-circuit respiratory calorimetry system to determine dietary energy utilization in broilers

**DOI:** 10.1093/jas/skaf369

**Published:** 2025-11-16

**Authors:** Hansuo Liu, Feng Zhao, Tiantian Sun, Changlin Guo, Yuming Wang, Jingjing Xie

**Affiliations:** The State Key Laboratory of Animal Nutrition and Feeding, Institute of Animal Science, Chinese Academy of Agricultural Sciences, Beijing, 100193, China; The State Key Laboratory of Animal Nutrition and Feeding, Institute of Animal Science, Chinese Academy of Agricultural Sciences, Beijing, 100193, China; The State Key Laboratory of Animal Nutrition and Feeding, Institute of Animal Science, Chinese Academy of Agricultural Sciences, Beijing, 100193, China; The State Key Laboratory of Animal Nutrition and Feeding, Institute of Animal Science, Chinese Academy of Agricultural Sciences, Beijing, 100193, China; The State Key Laboratory of Animal Nutrition and Feeding, Institute of Animal Science, Chinese Academy of Agricultural Sciences, Beijing, 100193, China; The State Key Laboratory of Animal Nutrition and Feeding, Institute of Animal Science, Chinese Academy of Agricultural Sciences, Beijing, 100193, China

**Keywords:** broiler, energy utilization, repeatability, respiratory calorimetry

## Abstract

Net energy (NE) is most precise measure of dietary energy for broilers, but its accurate determination requires a reliable respiratory calorimeter. This study developed a computer-controlled closed-circuit respiratory calorimeter to measure oxygen (O_2_) consumption, carbon dioxide (CO_2_) production, and energy utilization in broilers, and evaluated its accuracy and repeatability. Three groups of six closed-circuit calorimeters were randomly assigned to burn 100, 150, or 200 g of ethanol (purity ≥ 99.7%) to assess the accuracy of O_2_ usage and CO_2_ production. Subsequently, three groups of six calorimeters, each containing three male Arbor Acres broilers (body weight [BW] = 948 ± 14 g) as one experimental unit were used to test the repeatability of the chamber environment control, growth performance, and the determination of dietary energy utilization in broilers. Sensors automatically logged temperature, humidity, O_2_ and CO_2_ concentrations, O_2_ consumption, BW, and average daily gain (ADG). Data analysis was conducted using SAS 9.4 using the MEANS, GLM, and REG procedures. The relative ratios of actual O_2_ consumption and CO_2_ production to theoretical values (from complete ethanol combustion) ranged from 100.4% to 101.3% (*P* = 0.326), and 102.7% to 102.8% (*P *= 0.981), respectively, with all CV below 1.53%, demonstrating the system’s accuracy and precision in quantifying respiratory gas exchange. The CV of inter-group (CV_inter-group_) for temperature, humidity, and O_2_ concentration were 0.08% (*P *= 0.664), 1.14% (*P *= 0.726), and 0.09% (*P *= 0.203), respectively. The CV_inter-group_ for ADG, average daily feed intake (ADFI), and feed conversion ratio were 3.09% (*P *= 0.551), 2.24% (*P *= 0.580), and 2.11% (*P *= 0.364), respectively. The CV_inter-group_ for O_2_ consumption and CO_2_ production were 1.21% (*P *= 0.903) and 1.86% (*P *= 0.758), respectively, and both factors were linearly related to BW^0.70^ and ADFI (R^2^ ≥ 0.889, *P *< 0.010). The CV_inter-group_ for apparent metabolizable energy, total heat production, heat increment, NE, retained energy, NE deposited as protein or as fat ranged from 0.21% to 3.19% (0.236 ≤ *P * ≤  0.903). These results demonstrate the system’s high repeatability and precision in maintaining environment conditions and monitoring growth performance and energy partitioning in broilers. Thus, this system is a valuable tool for accurately evaluating energy utilization in broilers.

## Teaser

This study developed a computer-controlled closed-circuit respiratory calorimetry system and demonstrated its good accuracy and repeatability in measuring O_2_ consumption, CO_2_ production, and energy partitioning in broilers. These results highlight the system’s potential as a reliable and precise tool for consistent assessment of energy metabolism in broilers.

## Introduction

Dietary available energy represents the largest cost-factor in broiler production (Noblet et al., 2022). Net energy (NE), defined as apparent metabolizable energy (AME) minus heat increment (HI, total heat production minus fast heat production) is widely recognized as the most accurate estimate of available energy in feed for broilers (Noblet et al., 2010; Liu et al., 2017; Zuidhof, 2019). However various methods for determining NE vary in precsion, accuracy, and practicality.

Indirect respiratory calorimetry is widely used to determine total heat production (THP) by measurement of oxygen (O_2_) consumption and carbon dioxide (CO_2_) production in animals (Gerrits and Labussière, 2015). The reliability of THP and NE calculations therefore depends on the accuracy of these gas exchange measurements (Mtaweh et al., 2018). Conventional open-circuit indirect calorimetry method has been shown to produce substantial errors (up to 38%) in THP estimates (Walsberg and Hoffman, 2005). Although recent studies have investigated NE determination for poultry feed ingredients (Wu et al., 2019; Tay-Zar et al., 2024), detailed descriptions of respiratory calorimetry system design and data repeatability remain limited. Swick et al. (2013) reported CV of 5.5, 4.2, 4.6, and 3.7% for THP, respiratory quotient (RQ), fasting heat production (FHP), and NE respectively, using 12 closed-circuit calorimetry chambers for broilers fed a wheat-soybean meal-canola diet. Similarly, De Lange and Birkett (2005) reported that calorimetry techniques may yield inaccurate NE estimates due to analytical errors in measuring HI. Such errors may arise from factors including chamber leakage, inadequate environmental control (e.g., temperature, humidity, O_2_ and CO_2_ concentrations), and measurement errors in body weight (BW), O_2_ consumption, or CO_2_ production.

To address these limiations, a system that integrates respiratory calorimetry with advanced automatic control techniques is required to improve accuracy and repeatability. This study aimed to develop a computer-controlled closed-circuit respiratory calorimetry system and evaluate its repeatability in measuring O_2_ consumption, CO_2_ production, THP, HI, NE, and growth performance of broilers and maintaining environmental control. This work provides a methodological basis for partitioning the AME into HI and NE more accurately, thereby improving estimates of feed energy values and supporting optimization of feed cost and broiler performance.

## Materials and Methods

All experimental procedures related the use of live broilers were approved by the animal care and welfare committee of the Institute of Animal Sciences, Chinese Academy of Agricultural Sciences (Beijing, China; ethical approval code: IAS 2024-60).

### Experimental design

A full system test was conducted to assess the accuracy of O_2_ consumption and CO_2_ production, following procedures described by Gerrits and Labussière (2015). Three groups of six computer-controlled closed-circuit respiratory calorimetry systems (model CRS-1, Institute of Animal Science, Chinese Academy of Agricultural Sciences, Beijing) were used to burn 100, 150, or 200 g of ethanol (purity ≥ 99.7%; Sinopharm Chemical Reagent Co., Ltd, Shanghai, China) for groups 1, 2, and 3, respectively. Measured and relative O_2_ consumption and CO_2_ production per g of ethanol burned were compared with theoretical values calculated from complete ethanol combustion and the RQ was evaluated across the three groups.

Fifty-four 21-day-old male Arbor Acres broilers with similar BW (948 ± 14 g) were randomly assigned to three groups of six closed-circuit indirect respiratory calorimeters to assess repeatability of chamber environment control and the determination of dietary energy partition in broilers. Each chamber contained three broilers, which served as one experimental unit.

### Broilers management and experimental diet

From 22 to 28 d of age, broilers were provided with experimental diet and had free access to water (Table 1). Birds underwent an adaption period from 21 to 24 d of age and were tested from 25 to 28 d of age. Temperature, humidity, ventilation, and stocking density followed the management guidelines for Arbor Acres broilers. Diets were formulated to meet or exceed nutrient recommendations for broilers in China (MOA, 2004) (Table 2). All ingredients were crushed through a 2 mm sieve, thoroughly mixed, and pelleted (3 mm diameter × 3 mm length pellets) using a laboratory non-steam press pellet mill (Model SKJ 150, Funong machine Co. Zhengzhou, Henan, China).

**Table 1.** Ingredients and chemical composition of diets (as-fed basis, %)

Items	Starter and grower diet (day 1 to 21)	Experimental diet (day 22 to 28)
**Corn**	53.40	59.31
**Soybean meal**	35.38	29.67
**Corn gluten meal**	2.00	3.00
**Soybean oil**	4.51	4.08
**Dicalcium phosphate**	1.73	1.83
**Sodium chloride**	0.30	0.30
**Limestone**	1.07	0.74
**Premix^1^**	0.50	0.50
**L-lysine HCl**	0.50	0.28
**DL-methionine**	0.23	0.17
**L-threonine**	0.19	0.08
**L-valine**	0.14	0.02
**Broiler complex enzyme^2^**	0.03	0.00
**Phytase^3^**	0.02	0.02
**Total, %**	100	100
**Nutrient content, %^4^**		
**Dry matter**	88.25	87.97
**AME, kcal/kg**	3,055	3,086
**Crude protein**	22.16	20.30
**Calcium**	0.91	0.80
**Available phosphorus**	0.43	0.45

1 Supplied per kilogram of diets: vitamin A, 10,000 IU, vitamin D_3_, 4,000 IU, vitamin E, 55.0 IU, vitamin K_3_, 3.20 mg, thiamin, 3.0 mg, riboflavin, 7.0 mg, vitamin B_6_, 3.0 mg, vitamin B_12_, 16.0 µg, pantothenic acid, 15.0 mg, nicotinic acid, 50.0 mg, folic acid, 1.8 mg, biotin, 0.22 mg, choline chloride, 1,500 mg, Cu (as copper sulfate), 16.0 mg, Fe (as ferrous sulfate), 20 mg, Mn (as manganese sulfate), 120 mg, Zn (as zinc sulfate), 120 mg, I (as calcium iodate), 1.25 mg, Se (as sodium selenite), 0.30 mg.

2 Broiler complex enzyme (Beijing Challenge Bio-tech Co. Ltd, Beijing, China) provided 6,000 units/g of xylanase, 3,000 units/g of β-mannanase, 1,200 units/g of β-glucanase, and 100 units/g of cellulase.

3 Phytase (Beijing Challenge Bio-tech Co. Ltd, Beijing, China) provided enzyme activity 10,000 units per g.

4 Values were calculated values (air-dry basis) according to the China Feed Database (IASCAAS et al., 2024).

### Closed-circuit respiratory calorimetry to quantify O_2_ consumption and CO_2_ production

**Figure 1.**
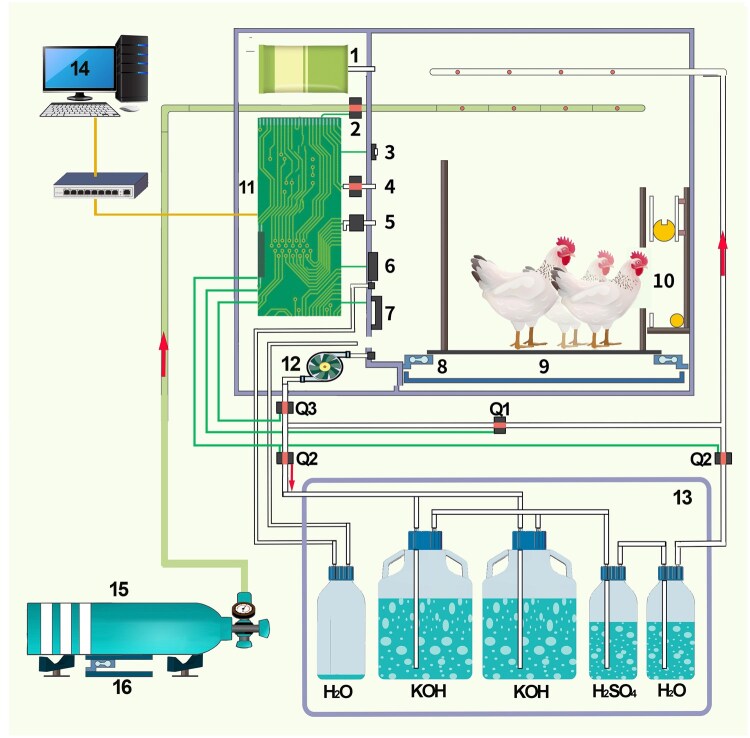
General design of closed-circuit respiratory calorimetry system. 1. Pressure-regulated bag; 2. Oxygen valve; 3. Temperature and humidity sensor; 4. Ventilation valve; 5. Differential pressure transmitter; 6. Refrigeration unit; 7. Heating device; 8. Weight sensor of bird; 9. Fecal tray; 10. Feeding devices; 11. Integrated circuit board; 12. Diaphragm pump; 13. Sealed box; 14. data-acquisition system; 15. Oxygen cylinder; 16. Weight sensor; Q1, Q2, Q3 are solenoid valves.

A computer-controlled closed-circuit calorimetry system (model CRS-1, Institute of Animal Science, Chinese Academy of Agricultural Sciences, Beijing) was used to automatically control and monitor the chamber environment (temperature, humidity, O_2_ and CO_2_ concentrations), record broiler BW, quantify O_2_ consumption, and collect CO_2_ (Figure 1). To achieve these functions, the system comprised four integrated subsystems: (1) the chamber system, (2) the chamber environment control system, (3) the O_2_ supply and consumption quantification system, and (4) the air filtration and CO_2_ absorption system. The chamber system consisted of an airtight chamber with a sealable door, housing a stainless-steel cage (75 cm length × 58 cm width × 50 cm height) suspended above a removable tray for excreta collection. Each cage contained a load cell sensor to continuously monitor BW of broilers. An automated feeding device programmed to dispense water, supply feed, and withdraw residual feed at the end of the bioassay. The chamber environmental control system was equipped with sensors for monitoring temperature and relative humidity (Model EE060, E + E Elektronik Ges.m.b.H., Engerwitzdorf, Australia) and O_2_ and CO_2_ concentrations (Model G1020, Wost technology Co. Ltd, Shenzhen, China). An air compression cooler and a ventilation heating pipe maintained temperature and humidity within specified ranges. The oxygen supply and consumption quantification system consisted of an O_2_ cylinder, a load cell sensor, and a solenoid valve. When chamber O_2_ concentration fell by >0.01% of the initial level, the solenoid valve automatically opened to release O_2_ from the cylinder into the chamber. The load cell sensor recorded the weight of the O_2_ cylinder every 10 S to calculate O_2_ consumption from weight change. Air filtration and CO_2_ absorption systems consisted of a one-way air pump, a 1 L sulfuric acid solution (0.70% vol/vol) to remove NH_3_, two 6 L KOH solution (350 g/kg) to absorb CO_2_. Air was pumped from the chamber and passed sequentially through sulfuric acid, KOH and deionized water solutions before returning to the chamber. These 4 subsystems automatically progressed from adaptation to quantification of O_2_ consumption and CO_2_ production of broilers, as controlled by dedicated software (copyright 2024SR0019052).

The procedure to quantify O_2_ consumption and CO_2_ production was described as follows. Prior to each bioassay, a bottle of 1 L sulfuric acid solution (0.70% vol/vol), two bottles of pre-weighed KOH solution and a bottle of 1 L deionized water were connected in sequence with silicon tubing, placed in a stainless-steel box (49.5 cm length × 43.5 cm width × 49.5 cm height) with a sealed door, and connected to the air inlet and outlet pipes of the box. The air inlet and outlet were connected to the outlet and inlet of the respirometry chamber, respectively, using silicon tubing, and the door was closed. Subsequently, an airtightness self-test procedure was initiated by the control software. O_2_ was injected into the chamber to raise the internal pressure to 300 Pa above atmospheric pressure, and the chamber was considered airtight if the differential pressure remained at ≥250 Pa for 30 min. Broilers were then placed in the cage with free access to diet and water during a 3-d adaptation period, while the chamber door remained open and conditions were maintained at 25 °C and 60% relative humidity. During adaption, air circulated without passing through the air filtration and CO_2_ absorption system, and all electrical components, except those used for temperature control, were turned off. Before starting the test, chamber setting (temperature, humidity, O_2_ concentration, differential pressure, feeding time and frequency, and test duration) were programmed in the control software. Feeder was emptied, refilled with a pre-weighted 3-d feed allotment. The chamber door was then sealed, and the bioassay commenced upon selecting “start” in the control software, continuing until the designated test duration was completed. Throughout the test, sensors continuously monitored chamber conditions, O_2_ and CO_2_ concentration, differential pressure, BW, and O_2_ consumption of broilers. Data were recorded and average BW, O_2_ consumption per kg BW^0.7^ and ADG were calculated using the control software. Upon completion of the bioassay, residual feed in the feeder was cleared automatically by opening the base plate. The air filtration and CO_2_ absorption system were deactivated, and the solenoid valve connected to the chamber was opened to allow atmospheric air exchange. Data collection was then stopped. The chamber door was opened, and the feeding device was weighed to calculate the feed intake. All excreta were collected into an aluminum foil box and stored at −20°C. After three consecutive 24-h bioassays in each chamber, the CO_2_-absorbing KOH solution was weighed and sampled to determine the total CO_2_ production during the test period. The CO_2_ absorbed in the KOH solution was quantified using BaCl_2_ precipitation method, as described by Wu et al. (2019). The density of CO_2_ (1.842 g/L, at 20 °C and 101.325 kN/m^2^) was used to convert weight (g) to volume (L).

### Determination of AME, THP, and NE

The dietary AME was determined based on the total feed intake and total excreta collected from broilers aged 25 to 28 d during three consecutive 24-h calorimetry bioassays. All excreta from each experimental unit were pooled, mixed and transferred to a forced air oven at 65 °C, and dried for 72 h. The O_2_ consumption and CO_2_ production were determined using the computer-controlled closed-circuit respiratory calorimetry system during the same three 24-h bioassays. The THP was calculated according to the formula published by Brouwer (1965) based on the volume of O_2_ consumed and CO_2_ produced. The RQ was calculated as ratio of volume of CO_2_ produced to volume of O_2_ consumed. The HI was calculated as the difference between THP and FHP. The dietary NE content (kcal/kg DM) was calculated as AME minus HI, expressed per kg of dietary DM.

### Chemical analysis

Samples were finely ground using a laboratory mill (model BJ-150, Deqing Baijie Electrical Co., Ltd, Zhejiang, China) and passed through a 0.42 mm mesh screen prior to chemical analysis. The DM content was determined following the AOAC (2007) method. The gross energy (GE) was measured by adiabatic calorimeter (Parr 6400, Parr Instrument Co., Moline, IL), with benzoic acid as the calibration standard. The crude protein (CP) content was determined by the AOAC (2007) method, using a Kjeldahl nitrogen analyzer (model KDY-9820, Shandong Haineng Scientific Instruments Co., Ltd, Dezhou, China).

### Calculation and statistical analysis

Dietary AME (kcal/kg DM) = (energy intake—energy output)/feed intake.

THP (kcal) = 3.866×O_2_ consumed (L) + 1.200 × CO_2_ exhaled (L) (Brouwer, 1965)

FHP (kcal/kg BW^0.70^ per day) = 107.55 (Noblet et al., 2015)

HI (kcal/kg DM) = (THP − FHP × BW^0.70^ × day)/FI (kg DM)

NE (kcal/kg DM) = AME − HI

Retained energy (RE, kcal/kg DM) = AME − THP/FI

RE as protein (RE_p_, kcal/kg DM) = RN × 6.25 × 5.70

RE as fat (RE_f_, kcal/kg DM) = RE—RE_p_

in which 6.25 is the protein equivalent of 1 g nitrogen, and 5.70 is the energy equivalent (kcal) of 1 g protein, as reported by Sharma et al. (2021).

The CV was calculated according to the formulas described by Jiang and Xia (2006):


$$\begin{array}{c} \mathrm{Total}\;\mathrm{CV}\;({\mathrm{CV}}_{\mathrm{total}})=\sqrt{\frac{\sum_{i=1}^{G}\sum_{j=1}^{N_{i}}\frac{1}{N}{\left(Y_{\mathrm{ij}}-\bar{Y}\right)}^{2}}{{\bar{Y}}^{2}}}\\ \begin{array}{c} CV\;\mathrm{of}\;\mathrm{inter}-\mathrm{group}\;({\mathrm{CV}}_{\mathrm{inter}-\mathrm{group}})=\sqrt{\frac{\sum_{i=1}^{G}\sum_{j=1}^{N_{i}}\frac{1}{N-G}{\left(Y_{\mathrm{ij}}-\bar{Y_{i}}\right)}^{2}}{{\bar{Y}}^{2}}}\\ CV\;\mathrm{of}\;\mathrm{intra}-\mathrm{group}\;({\mathrm{CV}}_{\mathrm{intra}-\mathrm{group}})=\sqrt{\frac{\sum_{i=1}^{G}\frac{N_{i}}{N}{\left(\bar{Y_{i}}-\bar{Y}\right)}^{2}}{{\bar{Y}}^{2}}} \end{array} \end{array}$$

in which Y_ij_ is the single observed data; $\bar{Y_{i}}$ is the mean of the ith group; $\bar{Y}$ is the mean of all observed data; G is the number of groups; N is the total number of observations; and N_i_ is the number of observations in the ith group.

Summary statistics for chamber environment variables (temperature, humidity, O_2_ concentration, and CO_2_ concentration), broiler performance (BW and ADG), energy partition (O_2_ consumption, CO_2_ production, AME, THP, HI, NE, RE, RE_p_, RE_f_), and energy utilization (AME/GE, NE/GE, RE/GE, RE_p_/GE, RE_f_/GE, NE/AME, THP/AME) were calculated using the MEANS procedure of SAS 9.4 (SAS Institute Inc., Cary, NC). Significant differences were identified using the Tukey honest significant difference test. Linear models of O_2_ consumption or CO_2_ production as function of ADFI and BW^0.70^ were developed using the REG procedure of SAS 9.4, with the following form: Y = β_1_ × ADFI + β_2_ × BW^0.70^, where Y represents O_2_ consumption or CO_2_ production, and β1 and β2 are regression coefficients. The adjusted R^2^ value was defined as 1 − error sum of squares/total sum of squares. Significance was set at *P *< 0.05, whereas 0.05 ≤ *P *< 0.10 was considered a tendency.

## Results

### O_2_ usage, CO_2_ production, and RQ during ethanol burning

The accuracy of O_2_ and CO_2_ analysis in the respiratory calorimetry system was evaluated using the ethanol burning test with three different ethanol quantities (100 g, 150 g, and 200 g). No significant differences were observed among the groups for the measured O_2_ usage and CO_2_ production per g of ethanol burned, nor for the relative O_2_ usage, CO_2_ production, or the RQ compared with the theoretical values calculated from complete ethanol combustion (Table 2). The relative O_2_ usage was 100.8, 100.4, and 101.3% across the three groups, and the relative CO_2_ production was 102.7, 102.8, and 102.7%, respectively. The RQ values remained consistent at 0.684, 0.687, and 0.681 across the groups. The CV for O_2_ usage, CO_2_ production, and RQ were all below 1.53% in the closed-circuit respiratory chambers across three groups.

**Table 2.** The accuracy of O_2_ and CO_2_ analysis in the closed-circuit respiratory calorimetry system based on the ethanol burning^1^

Item	Ethanol^2^ level, g						CV, %		
	100	150	200	SEM	*P*-value^3^		CV_intra-group_	CV_inter-group_	CV_total_
**O_2_ usage, L/g**	1.471	1.466	1.478	0.006	0.335		1.31	0.56	0.79
**CO_2_ production, L/g**	1.006	1.007	1.006	0.003	0.981		0.44	1.15	0.51
**Relative O_2_ usage, %^4^**	100.8	100.4	101.3	0.4	0.326		1.31	0.56	0.79
**Relative CO_2_ production, %^5^**	102.7	102.8	102.7	0.3	0.981		0.44	1.15	0.51
**Respiratory quotient^6^**	0.684	0.687	0.681	0.004	0.503		1.48	1.53	0.87

1 Data are presented as least squares means of six observations per treatment.

2 Ethanol (Sinopharm Chemical Reagent Co., Ltd, Shanghai, China) is reagent-grade anhydrous ethanol with a purity ≥99.7%.

3 Means were separated using Tukey honest significant difference test.

4 Relative O_2_ usage (%) = O_2_ measured (L) × 100%/(ethanol burned (g) × 1.4596 (L/g)).

5 Relative CO_2_ production (%) = CO_2_ measured (L) × 100%/(ethanol burned (g) × 0.9731 (L/g)).

6 Respiratory quotient = CO_2_ production (L/g)/O_2_ usage (L/g).

Abbreviations: CV_total_ = total CV; CV_inter-group_ = CV of inter-group; CV_intra-group_ = CV of intra-group.

### Monitored chamber environment in respiratory calorimetry

No significant differences were observed among the groups for temperature, relative humidity, O_2_ concentration, or CO_2_ concentration in the respiratory chambers (Table 3). The average environmental parameters were consistent across groups. Average temperature values ranged from 25.26 to 25.31 °C, with CV of 0.39, 0.08, and 0.36% for CV_intra-group_, CV_inter-group_, and CV_total_, respectively. Average relative humidity ranged from 56.96 to 58.25%, with CV of 5.97, 1.14, and 5.57% for CV_intra-group_, CV_inter-group_, and CV_total_, respectively. For O_2_ concentration, the averages ranged from 20.85 to 20.89%, with CV of 0.18, 0.09 and 0.18% for CV_intra-group_, CV_inter-group_, and CV_total_, respectively. For CO_2_ concentration, the averages ranged from 2,315 to 2,626 ppm across the three groups, with CV of 21.37, 5.32 and 20.22% for CV_intra-group_, CV_inter-group_, and CV_total_, respectively.

**Table 3.** Variation of environmental parameters in closed-circuit respiratory calorimetry system^1^

Item	Group^2^						CV, %		
	1	2	3	SEM	*P-*value^3^		CV_intra-group_	CV_inter-group_	CV_total_
**Temperature, °C**	25.30	25.26	25.31	0.04	0.664		0.39	0.08	0.36
**Relative humidity, %**	56.78	58.25	56.96	1.40	0.726		5.97	1.14	5.57
**O_2_ concentration, %**	20.85	20.89	20.89	0.02	0.203		0.18	0.09	0.18
**CO_2_ concentration, ppm**	2,553	2,626	2,315	218	0.584		21.37	5.32	20.22

1 Data are presented as least squares means of six observations per treatment.

2 Groups 1, 2, and 3 each consisted of six closed-circuit indirect calorimetry chambers, with three 25-day-old male Arbor Acres broilers per chamber.

3 Means are separated using Tukey honest significant difference test.

Abbreviations: CV_total_ = total CV; CV_inter-group_ = CV of inter-group; CV_intra-group_ = CV of intra-group.

### Growth performance of broilers during respiratory calorimetry

**Figure 2.**
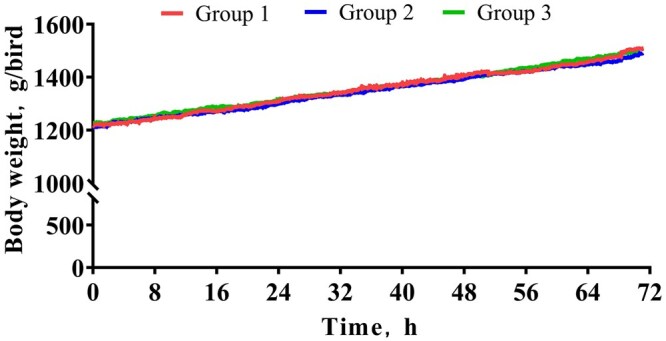
Body weight changes of broilers housed in closed-circuit respiratory calorimetry chambers across the three groups over a 72-h period (25 to 28 d of age). Groups 1, 2, and 3 each consisted of six closed-circuit indirect calorimetry chambers, with three 25-day-old male Arbor Acres broilers per chamber. Time is expressed in h.

Real-time monitoring of BW in broilers revealed similar slopes of growth curves from 25 to 28 d of age across the three groups (Figure 2). No differences were observed among the groups for initial BW (IBW), final body weight (FBW), metabiotic body weight (MBW), ADG, ADFI and feed conversion ratio (ADFI/ADG) in broilers aged 25 to 28 d (*P = *0.364 to 0.949; Table 4). The growth performance of broilers was similar across the three groups, with IBW ranging from 1,211 to 1,227 g, FBW from 1,485 to 1,510 g, MBW from 1,231 to 1,239 g, ADG from 91.1 to 97.6 g, ADFI from 117.6 to 124.2 g, and ADFI/ADG from 1.28 to 1.34. The CV_inter-group_ values for these parameters ranged from 0.28% to 3.09%, which were consistently lower than the CV_intra-group_ (range = 3.66 to 11.75%) and CV_total_ (range = 3.35 to 11.16%).

**Table 4.** Variation in growth performance of broilers in the closed-circuit respiratory calorimetry system from day 25 to 28 of age^1^

Item	Group^2^						CV, %		
	1	2	3	SEM	*P-*value^3^		CV_intra-group_	CV_inter-group_	CV_total_
**IBW on day 25, g**	1,219	1,211	1,227	25	0.905		5.03	0.53	4.62
**FBW on day 28, g**	1,510	1,485	1,498	31	0.856		5.10	0.67	4.70
**MBW, g**	1,239	1,231	1,238	18	0.949		3.66	0.28	3.35
**ADG, g/d**	97.6	91.8	91.1	4.5	0.551		11.75	3.09	11.16
**ADFI, g/d**	124.2	117.6	121.8	4.4	0.580		8.93	2.24	8.45
**ADG/ADFI, g/g**	0.78	0.78	0.75	0.03	0.364		6.08	2.11	5.94

1 Data are presented as least squares means of six observations per treatment.

2 Groups 1, 2, and 3 each consisted of six closed-circuit indirect calorimetry chambers, with three 25-day-old male Arbor Acres broilers per chamber.

3 Means are separated using Tukey honest significant difference test.

Abbreviations: IBW = initial body weight; FBW = final body weight; MBW = metabolic body weight, is equal to BW^0.70^; ADG = average daily gain; ADFI = average daily feed intake; CV_total_ = total CV; CV_inter-group_ = CV of inter-group; CV_intra-group_ = CV of intra-group.

### O_2_ consumption, CO_2_ production, and respiratory quotient

**Figure 3.**
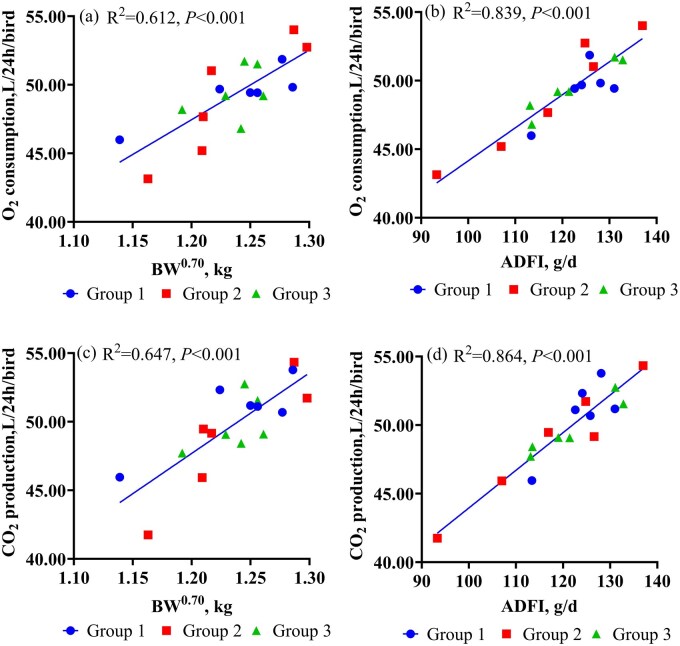
Linear regression of O_2_ consumption and CO_2_ production with BW^0.70^ and ADFI in broilers. Groups 1, 2, and 3 each consisted of six closed-circuit indirect calorimetry chambers, with three 25-day-old male Arbor Acres broilers per chamber. Regression analyses were performed using combined data from the three groups. The O_2_ consumption and CO_2_ production were strongly correlated with BW^0.70^ (O_2_ consumption, a, R^2^ = 0.612, *P* < 0.001; CO_2_ production, c, R^2^ = 0.647, *P* < 0.001) and ADFI (O_2_ consumption, b, R^2^ = 0.839, *P* < 0.001; CO_2_ production, d, R^2^ = 0.864, *P* < 0.001). Abbreviations: BW^0.70^ = metabolic body weight; ADFI = average daily feed intake. Panels: (a) Regression of O_2_ consumption on BW^0.7^. (b) Regression of O_2_ consumption on ADFI. (c) Regression of CO_2_ consumption on BW^0.7^. (d) Regression of CO_2_ consumption on ADFI.

The slopes and intercepts of linear regressions of O_2_ consumption and CO_2_ production (L/24h/bird) on MBW and ADFI did not differ among the three groups (*P *= 0.124 to 0.713 for slopes; *P *= 0.186 to 0.991 for intercept; Figure 3). Consequently, regression analyses were performed on pooled data from the three groups. The O_2_ consumption and CO_2_ production were strongly correlated with BW^0.70^ (R^2^ = 0.612, *P *< 0.001, Figure 3a; R^2^ = 0.647, *P *< 0.001, Figure 3c, respectively) and ADFI (R^2^ = 0.839, *P *< 0.001, Figure 3b; R^2^ = 0.864, *P *< 0.001, Figure 3d, respectively). The regression models were as follows: O_2_ consumption (L/24h/bird) = 22.14 × BW^0.70^ (kg) + 0.181 × ADFI (g) (R^2^ = 0.889, RMSE = 1.0), and CO_2_ production (L/24h/bird) = 16.25 × BW^0.70^ (kg) + 0.247 × ADFI (g) (R^2^ = 0.892, RMSE = 1.4).

No significant differences were detected among the three groups for daily O_2_ consumption per broiler, daily O_2_ consumption per kg of MBW, daily CO_2_ production per broiler, daily CO_2_ production per kg of MBW, or RQ (Table 5). Across groups, daily O_2_ consumption per broiler ranged from 49.22 to 49.69 L, daily O_2_ consumption per kg of MBW from 39.87 to 40.26 L, daily CO_2_ production per broiler from 49.98 to 50.85 L, daily CO_2_ production per kg of MBW from 40.47 to 41.21 L, and RQ from 1.01 to 1.02. The CV_intra-group_, CV_inter-group_, and CV_total_ for O_2_ consumption, CO_2_ production and RQ were all relatively low in the closed-circuit respiratory chambers. The CV_inter-group_ (range = 0.68 to 1.93%) was less than both CV_intra-group_ (range = 1.80 to 3.18%) and CV_total_ (range = 1.73 to 3.38%).

**Table 5.** Variation of O_2_ consumption, CO_2_ production and respiratory entropy in broilers between different groups^1^

Item	Group^2^						CV, %		
	1	2	3	SEM	*P*-value^3^		CV_intra-group_	CV_inter-group_	CV_total_
**Daily O_2_ consumption per broiler, L/d**	49.22	49.69	49.34	0.74	0.903		2.72	1.21	2.70
**Daily O_2_ consumption per kg of MBW, L/(kg BW^0.70^/d)**	39.87	40.26	39.94	0.60	0.893		2.44	1.24	2.49
**Daily CO_2_ production per broiler, L/d**	49.98	50.85	49.98	0.91	0.758		3.18	1.86	3.38
**Daily CO_2_ production per kg of MBW, L/(kg BW^0.70^/d)**	40.49	41.21	40.47	0.71	0.723		2.74	1.93	3.10
**Respiratory quotient^4^**	1.02	1.02	1.01	0.01	0.580		1.80	0.68	1.73

1 Data are presented as least squares means of six observations per treatment.

2 Groups 1, 2, and 3 each consisted of six closed-circuit indirect calorimetry chambers, with three 25-day-old male Arbor Acres broilers per chamber.

3 Means are separated using Tukey honest significant difference test.

4 Respiratory quotient = CO_2_ production (L/g)/O_2_ usage (L/g).

Abbreviations: MBW = metabolic body weight, is equal to BW^0.70^; CV_total_ = total CV; CV_inter-group_ = CV of inter-group; CV_intra-group_ = CV of intra-group.

### AME, THP, HI, NE, RE, and energy utilization efficiency

No significant differences were observed among the three groups for energetic values or energy utilization of the test diet (Table 6). Across groups, values ranged as follows: AME, 3,475 to 3,492 kcal/kg DM; THP, 2,023 to 2,089 kcal/kg DM; HI, 950 to 990 kcal/kg DM; NE, 2,490 to 2,542 kcal/kg DM; RE, 1,392 to 1,469 kcal/kg DM; RE_p_, 672 to 696 kcal/kg DM; and RE_f_, 720 to 772 kcal/kg DM. Similarly, energy utilization efficiency was also consistent across groups (Table 6), with AME/GE ranging from 75.13 to 75.49%, NE/GE from 53.83 to 54.96%, RE/GE from 30.09 to 31.75, RE_p_/GE from 14.52 to 15.05%, RE_f_/GE from 15.56 to 16.70%, NE/AME from 71.56 to 72.80% and THP/AME from 57.94 to 60.04%. The CV_inter-group_ for energetic values and energy utilization efficiency ranged from 0.21 to 3.19% and was less than the CV_intra-group_ (range = 1.89 to 6.18%) and CV_total_ (range = 1.72 to 6.07%) for all parameters except RE_f_ and RE_f_/GE, where the CV_intra-group_ and CV_total_ were 11.04 and 10.51%, respectively.

**Table 6.** Variation in energy utilization allocation in broilers among different groups^1^

Item	Group^2^						CV, %		
	1	2	3	SEM	*P*-value^3^		CV_intra-group_	CV_inter-group_	CV_total_
**Energy partitioning, kcal/kg DM**									
**AME**	3,492	3,480	3,475	28	0.903		1.89	0.21	1.72
**THP**	2,023	2,089	2,066	26	0.236		3.01	1.31	3.03
**HI**	950	990	970	17	0.270		4.06	1.67	4.04
**NE**	2,542	2,490	2,506	28	0.425		2.63	0.86	2.54
**RE**	1,469	1,392	1,409	37	0.332		6.18	2.31	6.07
**RE_p_**	696	672	684	12	0.377		4.11	1.43	3.99
**RE_f_**	772	720	726	34	0.510		11.04	3.19	10.51
**Energy utilization efficiency, %**									
**AME/GE**	75.49	75.24	75.13	0.60	0.905		1.89	0.21	1.72
**NE/GE**	54.96	53.83	54.17	0.60	0.423		2.63	0.86	2.54
**RE/GE**	31.75	30.09	30.47	0.80	0.333		6.18	2.31	6.07
**RE_p_/GE**	15.05	14.52	14.78	0.26	0.379		4.11	1.43	3.99
**RE_f_/GE**	16.70	15.56	15.69	0.74	0.508		11.04	3.19	10.51
**NE/AME**	72.80	71.56	72.08	0.46	0.207		1.53	0.70	1.55
**THP/AME**	57.94	60.04	59.47	0.88	0.252		3.53	1.49	3.54

1 Data are presented as least squares means of six observations per treatment.

2 Groups 1, 2, and 3 each consisted of six closed-circuit indirect calorimetry chambers, with three 25-day-old male Arbor Acres broilers per chamber.

3 Means are separated using Tukey honest significant difference test.

Abbreviations: AME = apparent metabolizable energy; DM = dry matter; NE = net energy; THP = total heat production; HI = heat increment; RE = retained energy; RE_f_ = retained energy as fat; RE_p_ = retained energy as protein; CV_total_ = total CV; CV_inter-group_ = CV of inter-group; CV_intra-group_ = CV of intra-group.

## Discussion

Respiratory calorimetry depends on precise measurements of gas exchange to accurately assess metabolic processes (Gerrits and Labussière, 2015). Assessing the recovery of O_2_ and CO_2_ is crucial for determining the accuracy of indirect calorimetry systems (Kaviani et al., 2018). The ethanol combustion test serves a well-established validation method, owing to the established stoichiometric respiratory quotient (RQ = 0.667) and predictable O_2_ consumption and CO_2_ production of ethanol (Gerrits and Labussière, 2015). In the present study, measured O_2_ consumption (1.471, 1.466, and 1.478 L) and CO_2_ production (1.006, 1.007, and 1.006 L) for the combustion of 1 g of ethanol across the three groups of closed-circuit indirect respiratory calorimeters closely matched the theoretical values of 1.460 L and 0.973 L, respectively, indicating the accuracy of the respiratory calorimetry system to measure gas exchange. Measured values relative to the theoretical values ranged from 100.4 to 101.3% for O_2_ consumption and from 102.7 to 102.8% for CO_2_ production across the three groups, within the acceptable limits of 97 to 103% reported by Mesgaran et al. (2020). These results demonstrate the robustness of the closed-circuit respiratory calorimetry system to accurately quantify respiratory gas exchange. Compared with previous respiratory calorimeter systems (Wu et al., 2019; Tay-Zar et al., 2024), the current achieved accurate quantification of O_2_ consumption through 2-hourly load cell sensor calibration, correcting for sensor deformation under prolonged oxygen cylinder stress.

Precise real-time monitoring and control of temperature, humidity, O_2_ and CO_2_ concentrations are crucial for accurate measurement of O_2_ consumption, CO_2_ production, and THP in respiratory experiments (Gerrits and Labussière, 2015; Barzegar et al., 2020). Brown-Brandl et al. (2004) reported that ambient air temperature significantly affects the logarithm of THP. In the present study, chamber temperature remained stable, with CV_intra-group_ and CV_inter-group_ below 0.39%. Such precision is critical for poultry trials, as minor temperature fluctuations can alter THP and feed-to-gain ratio (Bottje and Harrison, 1985), feed intake, carbohydrate metabolism, protein synthesis efficiency, fat deposition, and oxidative stress under hot and humid conditions (Kpomasse et al., 2021). The O_2_ concentration in the respiratory calorimetry chamber was also stable, with CV_intra-group_ and CV_inter-group_ below 0.18%. Maintaining a consistent O_2_ concentration during closed-circuit respiratory calorimetry reduces the influence of initial and final O_2_ concentration differences on actual O_2_ consumption by broilers. The CV_inter-group_ (1.14%) for humidity in the chamber was greater than that for temperature and O_2_ concentration, likely due to the slower response time of the humidity sensor, which results in delayed feedback regulation. Overall, the current closed-circuit respiratory calorimetry system demonstrated repeatable results in controlling temperature, humidity, and O_2_ delivery, meeting the requirements for studies on energy metabolism in broilers. The CO_2_ produced by the broilers was absorbed through reaction with potassium hydroxide to form potassium carbonate. The rate of CO_2_ removal decreased as the CO_2_ concentration in the chamber declined, maintaining fluctuations within 2,626 ppm, well below the 3,000 ppm threshold recommended in the Arbor Acre broiler management guidelines. These results indicate that this closed-circuit respiratory calorimetry system provided a stable environment for broilers through integrated sensor feedback control.

If broilers grow at suboptimal rates during indirect respiratory calorimetry, the measured O_2_ consumption and CO_2_ production may be biased, affecting the accuracy of THP calculation. While previous studies typically report only the initial and final BW of broilers during respiratory calorimetry (Tay-Zar et al., 2024), the present study employed real-time BW monitoring from the adaptation phase to the end of the respiratory calorimetry. Growth curves were consistent across groups. The CV_inter-group_ of FBW (0.67%), ADG (3.09%), ADFI (2.24%), and feed conversion rate (2.11%) were remarkably less than those reported by Swick et al. (2013), who observed CV_inter-group_ of 6.01%, 11.5%, and 8.1% for BW, ADG, and feed conversion rate, respectively, in broilers aged 25 to 27 d using 12 closed-circuit respiratory calorimetry chambers. The reduced CV values in the present study indicate superior and consistent environment control in the respiratory calorimetry chambers and successful automatic collection of BW data. The BW was automatically recorded every 10 s via the software, improving measurement accuracy and eliminating potential errors associated with manual weighing, such as stress-induced physiological response. These findings highlight the advantages of computer-assisted data acquisition, enabling real-time monitoring of BW in broilers throughout the respiratory calorimetry and enhancing both accuracy and efficiency.

Accurate and precise measurement of O_2_ consumption and CO_2_ production in broilers remains pivotal in respiratory calorimetry due to its substantial contribution to calculations of THP and RQ. Previous studies have reported that an acceptable relative bias for O_2_ consumption measurement ranged from 4.7 to 10.0% in broilers (Wagner et al., 1973; Davies et al., 1974) and showed similar levels for adult fowl in two indirect respiratory calorimetry systems (Boshouwers and Nicaise, 1981). Swick et al. (2013) reported a CV of RQ of 4.2% in broilers fed a wheat-soybean meal-meat-canola diet, measured using 12 closed-circuit calorimetry chambers. In the present study, daily O_2_ consumption per broiler, daily O_2_ consumption per kg of MBW, daily CO_2_ production per broiler, daily CO_2_ production per kg of MBW and RQ were consistent across the three groups, with intra-group CV ranging from 1.80 to 3.18% and inter-group CV ranging from 0.68 to 1.93%. This precision was achieved by calibrating the load cell sensor every 2 h to ensure accurate O_2_ consumption measurement. In addition, the accuracy of CO_2_ quantification in KOH solution was improved by ensuring complete rinsing of BaCO_3_ in the gravimetric method, with the completeness of rinsing verified by measuring the pH of the supernatant after centrifugation. Overall, the repeatability of the system exceeded conventional calorimeters, with CV_total_ below 3.38% for O_2_ consumption, CO_2_ production and RQ. These results demonstrate that the closed-circuit respiratory calorimeters used in this study provide reliable quantification of THP in poultry research.

Oxidation within the animal body comprises both basal metabolism and dietary oxidation, which can be quantified by O_2_ consumption and CO_2_ production. In the current study, BW^0.70^ was strong correlated with O_2_ consumption (R^2^ = 0.612) and CO_2_ production (R^2^ = 0.647). Both parameters were also highly correlated with ADFI (R^2^ = 0.839 for O_2_ consumption and R^2^ = 0.864 for CO_2_ production). These relationships indicate increases in BW and ADFI are associated with higher metabolic rates, which require greater O_2_ and result in higher CO_2_ production, and consequently, greater THP (Nascimento et al., 2017; Barzegar et al., 2020). Furthermore, linear regressions of O_2_ consumption or CO_2_ production against BW^0.70^ and ADFI suggest that the regression slope for BW^0.70^ represents the O_2_ consumption or CO_2_ production of broilers under fasting metabolism. Using the equation of Brouwer (1965): THP (kJ) = (3.866×oxygen consumption + 1.200 × carbon dioxide production) × 4.184, the calculated FHP and RQ were 440 kJ/kg BW^0.70^ and 0.73, respectively, which are closely aligned those reported by Noblet et al. (2015; 450 kJ/kg BW^0.70^ and 0.70). The close agreement between measured and reference values confirms the accuracy of the current closed-circuit respiratory calorimetry system for quantifying O_2_ consumption and CO_2_ production in broilers.

Determining energy partitioning in broilers requires accurate and precise measurement of AME, THP, FHP, and nitrogen retention. Although NE systems for broilers have been well developed (Wu et al., 2019; Barzegar et al., 2020; Sharma et al., 2021; Tay-Zar et al., 2024), the repeatability of measuring energy partitioning from AME to THP, NE, RE, RE_p_, and RE_f_ remains limited. In the present study, AME values were consistent across the three groups, with intra-group, inter-group and total CV below 1.89%, which were markedly less than the CV of 3.20 (Swick et al., 2013) and 4.80% (Tay-Zar et al., 2024) reported in previous studies. The ratio of THP to AME ranged from 57.94% to 60.04% among the three groups, a narrower range than the 50.00 to 57.66% reported previously, depending on the dietary AME, CP, and fat contents (Wu et al., 2019; Musigwa et al., 2021; Tay-Zar et al., 2024). Moreover, the intra-group, inter-group, and total CV for THP/AME were below 3.54%, considerably lower than 5.50% reported by Swick et al. (2013). These results further support the reliability and precision of the current closed-circuit respiratory calorimetry system for THP measurement. The NE values in the present study ranged from 2,490 to 2,542 kcal/kg DM across the three groups, with intra-group, inter-group, and total CV below 2.63%, substantially lower than those reported by Swick et al. (2013, 3.70%) and Tay-Zar et al. (2024, 6.2%). Correspondingly, the intra-group, inter-group and total CV for AME/GE, NE/GE, and NE/AME were all below 2.63%. Additionally, the determined RE, RE_p_, RE_f_, RE/GE, RE_p_/GE, and RE_f_/GE values were consistent across the three groups, with inter-group CV below 3.19%. Collectively, these findings demonstrate that the closed-circuit respiratory calorimeter used in the present study provides a precise and repeatable means of determining energy partitioning and utilization efficiency in broilers.

## Conclusion

The computer-controlled closed-circuit respiratory calorimetry system demonstrated high precision in maintaining environmental conditions, real-time monitoring of broiler BW, and accurate measurement of O_2_ consumption and CO_2_ production. These gas exchange variables were strong linear relationships with BW^0.70^ and ADFI (R^2^ ≥ 0.889). The system provided repeatable results in environmental control, broiler growth performance, and energy partitioning. Overall, these results support the system as a reliable tool for accurately assessing energy metabolism in broilers.

## Abbreviations:

ADFI: average daily feed intake
ADG: average daily gain
AME: apparent metabolizable energy
BW: body weight
CO_2_: carbon dioxide
CP: crude protein
CV_inter-group_: CV of inter-group
CV_intra-group_: CV of intra-group
CV_total_: total CV
DM: dry matter
FBW: final body weight
FHP: fasting heat production
GE: gross energy
HI: heat increment
IBW: initial body weight
MBW: metabolic body weight, is equal to BW^0.70^
MCP: metabolizable crude protein
ME: metabolizable energy
NE: net energy
RE: retained energy
O_2_: oxygen
RE_f_: retained energy as fat
RE_p_: retained energy as protein
RN: retained nitrogen
RQ: respiratory quotient
THP: total heat production
